# Supplementary material for: Genome-Wide Population Structure and Selection Signatures of Yunling Goat Based on RAD-seq
Source: Animals (Basel). 2022 Sep 13;12(18):2401. doi: 10.3390/ani12182401 (PMC9495202; doi:10.3390/ani12182401)
Supplement: Supplementary file 1 [file animals-12-02401-s001.zip › animals-1903391-supplementary.pdf]

animals

Supplementary Files

Table S1. Statistical table of sequencing quality.

Sample ID	Number of Bases in Raw Data (bp)	Number of High-Quality Reads	High Quality Total Number of Bases (bp)	Clean GC (%)	Clean Q30(%)
YR192	5,628,596,183	18,961,533	5,628,597,706	45.05	93.53
YR218	5,519,121,282	18,654,356	5,519,122,294	44.25	93.5
YR195	5,560,966,735	18,860,667	5,560,967,882	44.82	93.75
YR196	5,808,310,757	19,699,840	5,808,311,991	44.46	93.59
NBY122	6,360,894,381	21,647,195	6,360,895,994	44.2	93.08
YR199	5,464,092,951	18,531,712	5,464,093,794	44.48	93.48
NBY120	4,403,955,259	14,987,258	4,403,956,030	43.87	92.79
YR217	5,080,103,413	17,170,355	5,080,104,381	44.23	93.42
YR215	5,273,919,860	17,826,424	5,273,922,069	44.77	93.5
YR214	5,474,823,403	18,504,655	5,474,824,837	44.28	93.42
YR204	6,084,661,216	20,707,555	6,084,662,740	44.54	93.78
YR205	5,145,131,346	17,509,144	5,145,132,265	44.7	93.51
YR201	5,845,698,197	19,894,098	5,845,700,097	44.38	93.62
YR203	5,963,269,017	20,294,702	5,963,269,978	44.64	93.48
YR208	4,500,854,653	15,318,079	4,500,855,399	44.87	93.1
YR209	4,895,881,174	16,660,272	4,895,881,844	44.56	93.44
NBY56	4,351,834,739	14,609,742	4,351,835,720	43.61	93.5
NBY55	5,417,061,925	18,185,514	5,417,063,388	43.76	93.59
NBY53	5,185,489,298	17,525,938	5,185,490,909	43.7	93.66
NBY52	4,810,390,077	16,258,168	4,810,391,046	43.79	93.66
YR164	5,345,557,096	18,192,014	5,345,558,025	44.64	93.2
YR166	7,110,645,970	24,198,128	7,110,647,778	44.54	93.5
NBY59	6,230,630,060	20,986,849	6,230,631,253	43.65	93.83
NBY58	4,665,598,374	15,715,347	4,665,599,190	44.06	93.61
NBY35	5,949,231,363	20,245,441	5,949,232,649	44.3	93.06
NBY36	5,704,554,788	19,412,530	5,704,555,692	44.11	93.52
YR75	5,698,853,960	19,393,560	5,698,854,858	43.98	93.45
YR76	5,246,247,665	17,852,983	5,246,248,382	44.22	93.53
NBY32	6,721,582,482	22,873,519	6,721,583,821	44.27	93.58
YR79	5,730,835,013	19,370,629	5,730,836,806	44.12	93.28
NBY38	6,020,137,131	20,486,955	6,020,138,449	44	93.57
NBY23	5,959,960,140	20,282,108	5,959,961,993	43.95	93.53
NBY105	5,881,878,263	19,747,351	5,881,880,173	43.91	92.88
NBY107	5,573,445,328	18,712,476	5,573,446,851	43.66	92.77
NBY100	5,339,046,194	18,046,716	5,339,047,375	43.61	92.93
NBY101	4,639,218,162	15,680,771	4,639,218,925	43.75	92.96
NBY103	5,391,703,631	18,224,165	5,391,705,205	43.65	92.97
NBY109	5,522,309,231	18,602,570	5,522,310,216	43.76	92.93
NBY40	5,954,972,798	20,263,417	5,954,973,469	43.89	93.84
NBY42	6,249,514,703	21,122,105	6,249,516,304	43.73	93.6
NBY44	5,758,921,575	19,463,931	5,758,923,706	43.58	93.7
YR178	7,171,270,727	24,405,296	7,171,271,732	45.09	93.78
YR229	5,113,855,170	17,225,722	5,113,856,214	44.55	93.64
NBY48	5,856,488,600	19,793,818	5,856,489,741	43.61	93.6
YR174	6,527,795,444	22,216,495	6,527,797,253	45.22	93.6
YR172	7,389,489,608	25,147,681	7,389,490,706	44.98	93.21
YR173	5,529,061,555	18,816,414	5,529,062,403	44.93	93.56
YR168	6,326,484,649	21,533,358	6,326,486,361	45.63	93.43
NBY27	5,259,553,382	17,898,462	5,259,554,462	44.45	93.34
NBY22	6,091,587,719	20,659,853	6,091,589,009	44.3	93.41

YR66	4,360,920,293	14,840,653	4,360,921,151	44.42	93.31
NBY20	5,675,840,966	19,249,452	5,675,842,399	43.96	92.99
NBY29	5,656,174,628	19,248,199	5,656,176,114	43.95	93.66
NBY112	5,353,417,512	18,156,638	5,353,418,547	43.89	93.13
NBY110	5,189,127,768	17,480,349	5,189,128,926	43.7	93.17
NBY117	5,296,954,828	17,964,830	5,296,955,572	43.89	92.84
NBY115	4,839,455,873	16,413,520	4,839,457,191	44.22	92.33
NBY114	5,991,423,411	20,321,081	5,991,424,826	44.05	92.87
NBY118	5,444,961,632	18,530,435	5,444,963,645	44.02	92.91
YR148	5,419,017,632	18,378,691	5,419,018,939	44.43	93.47
YR143	5,139,168,845	17,312,130	5,139,170,144	44.23	93.32
YR142	5,826,118,827	19,560,350	5,826,120,232	44.47	93.21
YR140	6,667,180,969	22,385,259	6,667,184,031	44.7	93.29
YR147	5,066,631,211	17,067,563	5,066,632,408	44.25	93.54
NBY13	7,633,683,250	25,628,825	7,633,685,834	44.27	93.34
NBY11	6,908,873,021	23,194,816	6,908,875,371	44.11	93.48
NBY10	6,034,551,365	20,396,787	6,034,553,584	44.08	93.51
NBY17	6,004,590,859	20,363,785	6,004,592,090	43.9	93.68
NBY16	6,075,167,488	20,464,811	6,075,169,268	44.03	93.69
NBY14	5,753,151,347	19,380,005	5,753,152,973	44.14	93.47
NBY19	6,021,112,883	20,420,362	6,021,114,399	43.98	93.45
YR155	6,560,411,045	22,250,019	6,560,412,664	44.83	92.8
YR157	7,987,212,505	27,089,577	7,987,214,134	44.8	93.22
YR152	8,467,372,755	28,718,050	8,467,375,088	44.84	93.27
YR158	6,471,771,054	22,024,942	6,471,773,571	44.34	93.36
YR193	6,227,752,180	20,979,507	6,227,753,777	44.55	93.81
NBY08	6,406,384,794	21,653,878	6,406,386,739	44.02	93.5
NBY05	5,799,126,933	19,601,064	5,799,129,533	44.34	93.53
NBY07	5,703,041,362	19,275,775	5,703,042,987	43.7	93.45
NBY01	5,944,245,652	20,091,666	5,944,247,458	43.92	93.48
YR198	5,560,718,298	18,860,739	5,560,720,114	45.2	93
YR212	4,794,971,751	16,317,938	4,794,972,595	44.98	93.56
NBY125	6,107,065,464	20,783,364	6,107,066,610	44.36	92.53
YR211	4,935,583,045	16,795,610	4,935,583,720	44.27	93.53
YR123	4,980,535,173	16,834,544	4,980,537,075	43.94	93.44
YR242	5,181,915,765	17,633,641	5,181,917,208	43.87	93.47
YR245	4,784,130,298	16,280,379	4,784,130,950	44.09	93.32
NBY124	6,020,123,248	20,486,976	6,020,124,228	43.86	92.98
NBY92	6,485,573,543	22,070,239	6,485,574,408	43.73	93.03
NBY90	6,303,352,897	21,449,174	6,303,353,579	43.77	93.66
NBY97	5,066,441,948	17,125,033	5,066,444,028	43.88	92.98
NBY95	5,451,208,697	18,424,993	5,451,209,873	43.64	92.92
NBY94	5,679,716,862	19,328,523	5,679,717,606	44.19	93.13
NBY71	5,314,216,603	18,021,991	5,314,217,260	44.13	93.53
NBY73	6,239,562,228	21,232,778	6,239,564,148	44.1	93.65
YR132	4,997,413,308	16,891,633	4,997,414,579	44.32	93.36
NBY85	4,680,809,922	15,927,728	4,680,810,955	43.39	93.81
YR131	4,791,167,056	16,193,870	4,791,168,202	44.04	93.31
NBY83	5,521,422,090	18,788,631	5,521,422,759	43.8	93.53
NBY88	5,900,778,562	20,079,111	5,900,779,347	43.95	93.24
YR238	3,889,399,248	13,396,828	3,889,404,893	45.02	92.45
YR235	5,390,566,604	18,281,967	5,390,567,801	44.09	93.41
YR237	5,006,414,804	16,978,862	5,006,415,996	44.2	92.91
YR230	4,872,425,784	16,524,650	4,872,426,966	44.55	93.54
NBY69	3,968,621,857	13,459,354	3,968,622,951	44.46	93.03
NBY63	5,194,643,840	17,616,323	5,194,644,927	43.81	93.58
NBY61	5,448,532,340	18,477,045	5,448,533,133	44.14	93.78

NBY87	5,329,746,412	18,135,782	5,329,747,048	43.66	93.77
YR135	5,177,236,479	17,499,483	5,177,238,275	44.36	93.35
YR187	6,164,319,745	20,695,333	6,164,321,783	44.83	93.53
YR186	8,275,603,627	27,973,373	8,275,607,120	44.75	93.58
YR184	5,449,779,587	18,421,074	5,449,780,599	44.77	93.66
YR183	5,685,848,367	19,218,112	5,685,849,585	44.77	93.55
YR181	6,292,972,714	21,270,141	6,292,974,722	44.63	93.69
YR180	6,212,572,632	20,999,275	6,212,575,010	44.95	93.53
YR226	5,595,478,742	18,785,882	5,595,480,250	44.95	93.25
YR227	4,335,287,955	14,603,785	4,335,289,130	44.36	93.35
YR224	5,596,863,762	18,789,675	5,596,865,556	43.98	93.38
YR223	11,204,659,589	37,869,688	11,204,663,011	44.03	93.47
YR189	6,010,752,618	20,180,200	6,010,754,100	44.67	93.47

Notes：

Sample ID：Sample number.

YR：Yunling Goat.

NBY：Nubian Goat.

Table S2. Statistical table of the results of genome Alignment.

Sample ID	Mapped Ratio(%)	Proper Ratio(%)	Average Insert Size	Average Depth	Genome Coverage(1X)(%)	Genome Coverage(5X)(%)
YR192	99.92	95.22	382.4	1.92	21.53	13.16
YR218	99.93	95.08	381.8	1.89	22.03	13.35
YR195	99.93	95.33	380.4	1.9	21.48	13.15
YR196	99.93	95.22	380	1.99	21.94	13.63
NBY122	99.93	95.29	374.4	2.17	22.6	14.31
YR199	99.93	94.84	381.1	1.87	21.32	13
YR211	99.93	95.1	380.7	1.69	21.17	12.25
NBY90	99.93	95.69	375	2.16	21.83	14.24
YR217	99.91	95.09	382.4	1.74	21.28	12.45
YR215	99.93	94.93	381.5	1.8	21.59	12.71
YR214	99.93	94.98	382.4	1.87	21.88	13.16
YR204	99.93	94.78	379.2	2.08	22.16	13.99
YR205	99.92	94.83	380.6	1.76	21.34	12.43
YR201	99.93	94.98	379.3	2	21.83	13.62
YR203	99.93	94.62	379.5	2.04	22.03	13.79
YR208	99.94	95.23	378.3	1.54	20.92	11.5
YR209	99.93	95.1	380.8	1.67	20.98	12.03
NBY56	99.92	95.14	379.2	1.49	19.13	10.93
NBY55	99.91	95.15	379.2	1.85	20.72	12.75
NBY53	99.9	95.13	377.1	1.77	20.3	12.34
YR168	99.93	95.38	376.9	2.16	22.57	14.15
YR164	99.93	95.48	380.1	1.83	21.19	12.73
YR166	99.92	95.67	380.3	2.43	23.04	15.24
NBY59	99.91	95.26	378.4	2.13	21.54	13.94
NBY58	99.93	95.15	378.4	1.6	19.64	11.47
NBY35	99.92	95.46	369.3	2.03	20.87	13.4
NBY36	99.92	95.52	369.2	1.95	20.37	12.93
YR75	99.93	95.23	380.9	1.95	21.68	13.36
YR76	99.93	95.4	381	1.79	21.22	12.7
NBY32	99.91	95.28	369.5	2.3	21.34	14.14
YR79	99.92	94.83	382.6	1.96	21.7	13.4
NBY38	99.91	95.45	369.6	2.06	20.55	13.24
YR66	99.93	95.06	381.3	1.49	19.42	10.78
NBY105	99.92	95.45	379.1	2.01	22.27	13.77
NBY107	99.92	95.43	379.4	1.91	21.72	13.17
NBY100	99.9	95.54	376.2	1.82	21.88	13.25
NBY101	99.92	95.52	377.4	1.59	20.59	11.7
NBY103	99.91	95.4	377	1.84	21.74	13.16
NBY109	99.92	95.42	378	1.89	21.86	13.24
NBY40	99.91	95.41	375.1	2.04	21.48	13.73
NBY42	99.92	95.69	377.6	2.14	21.24	13.65
NBY44	99.92	95.69	377.3	1.97	20.89	13.18
YR178	99.93	95.38	379.3	2.45	23.19	15.38
YR229	99.93	94.68	383.9	1.75	21.23	12.39
NBY48	99.91	95.81	377.2	2	21.1	13.39
YR174	99.92	95.16	379.4	2.23	22.32	14.29
YR172	99.93	95.07	379.6	2.53	23.43	15.69
YR173	99.92	94.99	379.7	1.89	21.31	12.96
NBY52	99.92	95.29	377.5	1.64	19.62	11.56
NBY27	99.92	95.11	369.5	1.8	19.67	12.12
NBY22	99.92	95.18	369.8	2.08	20.88	13.5
NBY23	99.91	95.3	369	2.04	20.79	13.41
NBY20	99.92	95.5	370.6	1.94	20.27	12.77

NBY29	99.92	95.39	369.3	1.93	20.2	12.81
NBY112	99.93	95.35	374.9	1.83	21.89	13.2
NBY110	99.93	95.31	377.8	1.77	21.48	12.78
NBY117	99.93	95.45	375.7	1.81	21.63	12.99
NBY115	99.93	95.31	375.9	1.65	20.95	12.01
NBY114	99.92	95.33	375	2.05	22.62	14.17
NBY118	99.92	95.34	374.5	1.86	21.65	13.03
YR148	99.92	95.04	381.9	1.85	21.11	12.71
YR143	99.92	95.03	383.8	1.76	20.76	12.24
YR142	99.92	95.1	384.5	1.99	21.82	13.46
YR140	99.92	95.47	383.2	2.28	22.74	14.63
YR147	99.93	95.16	383.9	1.73	20.7	12.21
NBY13	99.91	95.52	373.3	2.61	22.2	15.27
NBY11	99.92	95.32	373.2	2.36	21.59	14.43
NBY10	99.92	95.36	371.2	2.06	20.63	13.3
NBY17	99.91	95.37	370.5	2.05	20.59	13.24
NBY16	99.91	95.56	372.4	2.08	20.63	13.34
NBY14	99.9	95.07	372.5	1.97	20.31	12.89
NBY19	99.91	95.13	370.6	2.06	20.67	13.25
YR155	99.93	95.38	381.2	2.24	22.51	14.39
YR157	99.93	95.82	381.1	2.73	23.81	16.27
YR152	99.93	95.52	381.2	2.89	24.08	16.61
YR158	99.92	95.33	379.6	2.21	22.56	14.52
YR193	99.93	95.23	382.2	2.13	22.29	14.23
NBY08	99.92	95.67	371.5	2.19	20.9	13.72
NBY05	99.91	95.46	371.4	1.98	20.32	12.88
NBY07	99.91	95.27	371.9	1.95	20.08	12.72
NBY01	99.91	95.36	371	2.03	20.69	13.3
YR198	99.93	94.99	380.2	1.9	21.61	13.06
YR212	99.93	94.87	380.2	1.64	21	11.89
NBY120	99.92	95.1	375.3	1.51	20.09	11.12
YR123	99.92	95.21	382.3	1.7	20.74	12.19
YR242	99.92	94.97	381.2	1.77	21.31	12.64
YR245	99.93	94.74	380.7	1.64	20.87	11.97
NBY124	99.92	95.54	375.8	2.06	22	13.61
NBY92	99.93	95.63	375.7	2.22	22.78	14.64
NBY125	99.93	95.37	375.4	2.09	22.41	13.93
NBY97	99.91	95.4	376.9	1.73	21.27	12.51
NBY95	99.92	95.64	377.3	1.86	21.79	13.25
NBY94	99.93	95.76	375.1	1.94	22.22	13.69
NBY71	99.93	95.35	376	1.82	20.53	12.6
NBY73	99.92	95.45	374.7	2.13	21.66	13.97
YR132	99.92	95.3	382.5	1.71	20.67	12.14
NBY85	99.92	95.42	375.9	1.6	19.32	11.35
YR131	99.91	95.1	383.4	1.64	20.23	11.71
NBY83	99.93	95.72	375.4	1.89	20.76	12.91
NBY88	99.93	95.58	375.9	2.02	21.31	13.54
YR238	99.91	95.98	357.9	1.33	22.7	10.71
YR235	99.93	95.15	381.2	1.84	21.92	13.18
YR237	99.93	95.18	381.7	1.71	21.43	12.49
YR230	99.93	94.59	381.7	1.67	20.85	11.96
NBY69	99.92	95.35	375.9	1.36	18.56	10.14
NBY63	99.92	95.3	376.4	1.78	20.35	12.32
NBY61	99.92	95.24	375.9	1.86	20.83	12.9
NBY87	99.92	95.42	375.9	1.82	20.41	12.52
YR135	99.92	95.21	382.4	1.77	20.92	12.45
YR187	99.93	94.99	384.1	2.11	22.06	13.88

YR186	99.93	94.67	381.3	2.83	23.77	16.54
YR184	99.93	95.49	381.1	1.86	21.48	13.09
YR183	99.9	95.08	382	1.94	21.48	13.19
YR181	99.92	95.12	381.7	2.15	22.3	14.25
YR180	99.92	95.02	381.4	2.12	22.21	14.05
YR226	99.93	94.37	384.4	1.91	21.82	13
YR227	99.93	94.51	384.6	1.48	19.87	10.88
YR224	99.92	94.65	385.5	1.91	21.76	13.15
YR223	99.93	96.24	383.6	3.83	25.58	19.08
YR189	99.92	95.49	384	2.05	21.87	13.77

Notes：

Sample ID：Sample number.

YR：Yunling Goat.

NBY：Nubian Goat.

Mapped Ratio：Percentage of the number of Clean Reads in the genome aligned to all Clean Reads.

Proper Ratio：The percentage of Clean Reads that were aligned to the reference genome and were in line with the length of the sequencing fragment by double-ended sequencing sequences.

Average Insert Size：Sequencing library size is the inserted fragment size after comparing the sequencing double-ended data with the genome.

Average Depth：Average sequencing depth is the ratio between the total number of bases in the previous genome and the total length of the reference genome.

Genome Coverage(1X)(%)：Percentage of total genome length covered by at least one Reads.

Genome Coverage(5X)(%)：The percentage of the total length of the genome covered by at least five Reads.

Table S3. Statistical table of SNP.

Sample ID	SNP Number	Transition	Transversion	Ti/Tv	Heterozygosity Number	Homozygosity Number
NBY01	1,813,943	1,213,781	598,499	2.03	838,321	975,622
NBY05	1,741,997	1,165,426	575,088	2.03	785,496	956,501
NBY07	1,732,393	1,161,156	569,802	2.04	801,487	930,906
NBY08	1,817,898	1,216,452	599,829	2.03	843,201	974,697
NBY10	1,788,527	1,199,989	586,975	2.04	833,231	955,296
NBY100	1,924,026	1,290,415	631,855	2.04	960,406	963,620
NBY101	1,771,619	1,188,873	581,164	2.05	825,725	945,894
NBY103	1,910,355	1,281,013	627,611	2.04	925,963	984,392
NBY105	1,965,732	1,315,735	648,163	2.03	946,324	1,019,408
NBY107	1,900,764	1,275,082	624,013	2.04	913,055	987,709
NBY109	1,932,334	1,291,365	639,235	2.02	915,211	1,017,123
NBY11	1,794,780	1,194,050	599,242	1.99	671,136	1,123,644
NBY110	1,874,916	1,257,811	615,531	2.04	887,449	987,467
NBY112	1,921,582	1,283,653	636,189	2.02	899,946	1,021,636
NBY114	1,996,636	1,334,721	660,071	2.02	921,763	1,074,873
NBY115	1,754,930	1,174,708	578,801	2.03	717,524	1,037,406
NBY117	1,903,610	1,276,112	625,747	2.04	906,505	997,105
NBY118	1,908,766	1,277,193	629,808	2.03	907,449	1,001,317
NBY120	1,739,047	1,164,658	572,916	2.03	803,686	935,361
NBY122	2,022,500	1,345,137	675,452	1.99	936,124	1,086,376
NBY124	1,963,924	1,314,035	648,108	2.03	953,482	1,010,442
NBY125	2,013,776	1,345,417	666,502	2.02	980,053	1,033,723
NBY13	1,982,714	1,326,775	654,096	2.03	967,878	1,014,836
NBY14	1,759,231	1,178,098	579,564	2.03	815,812	943,419
NBY16	1,794,368	1,203,891	588,885	2.04	844,622	949,746
NBY17	1,812,407	1,212,639	598,099	2.03	848,118	964,289
NBY19	1,799,738	1,204,637	593,527	2.03	847,906	951,832
NBY20	1,752,614	1,175,680	575,405	2.04	828,177	924,437
NBY22	1,819,338	1,212,578	605,187	2	812,925	1,006,413
NBY23	1,816,052	1,216,179	598,271	2.03	842,039	974,013
NBY27	1,678,175	1,118,467	558,157	2	736,699	941,476
NBY29	1,745,661	1,171,690	572,468	2.05	813,669	931,992
NBY32	1,879,076	1,252,447	624,922	2	877,341	1,001,735
NBY35	1,804,824	1,208,181	595,060	2.03	830,335	974,489
NBY36	1,775,488	1,188,324	585,532	2.03	830,440	945,048
NBY38	1,768,288	1,185,406	581,384	2.04	812,354	955,934
NBY40	1,871,133	1,251,549	617,871	2.03	871,074	1,000,059
NBY42	1,862,364	1,243,343	617,300	2.01	857,635	1,004,729
NBY44	1,794,625	1,202,897	590,199	2.04	820,122	974,503
NBY48	1,832,989	1,226,237	605,151	2.03	849,480	983,509
NBY52	1,679,592	1,124,315	553,830	2.03	742,888	936,704
NBY53	1,750,664	1,174,610	574,596	2.04	807,958	942,706
NBY55	1,803,596	1,205,241	596,766	2.02	835,577	968,019
NBY56	1,631,169	1,095,517	534,296	2.05	740,446	890,723
NBY58	1,662,274	1,111,672	549,195	2.02	732,225	930,049
NBY59	1,905,537	1,273,757	629,985	2.02	916,190	989,347
NBY61	1,820,964	1,211,331	607,931	1.99	824,700	996,264
NBY63	1,761,294	1,176,826	582,946	2.02	813,499	947,795
NBY69	1,560,655	1,045,477	513,837	2.03	689,749	870,906
NBY71	1,757,310	1,175,006	580,842	2.02	789,773	967,537
NBY73	1,907,023	1,272,887	632,371	2.01	912,704	994,319
NBY83	1,775,812	1,192,182	582,181	2.05	822,268	953,544
NBY85	1,648,297	1,105,906	540,998	2.04	731,778	916,519

NBY87	1,757,958	1,181,398	575,061	2.05	810,713	947,245
NBY88	1,849,292	1,239,095	608,559	2.04	859,763	989,529
NBY90	1,929,084	1,291,676	635,670	2.03	926,567	1,002,517
NBY92	2,027,893	1,355,839	670,222	2.02	988,568	1,039,325
NBY94	1,958,543	1,313,783	643,073	2.04	966,881	991,662
NBY95	1,902,710	1,273,421	627,597	2.03	913,240	989,470
NBY97	1,852,453	1,240,364	610,438	2.03	890,416	962,037
YR123	1,794,362	1,200,189	592,652	2.03	780,015	1,014,347
YR131	1,706,949	1,143,468	562,048	2.03	701,554	1,005,395
YR132	1,732,048	1,154,705	575,925	2	670,252	1,061,796
YR135	1,740,474	1,161,114	577,992	2.01	658,298	1,082,176
YR140	2,002,017	1,333,907	666,334	2	910,636	1,091,381
YR142	1,890,523	1,260,056	628,748	2	834,799	1,055,724
YR143	1,714,480	1,139,804	573,302	1.99	599,688	1,114,792
YR147	1,716,284	1,143,999	571,028	2	629,618	1,086,666
YR148	1,788,635	1,190,745	596,387	2	688,614	1,100,021
YR152	2,130,703	1,416,021	712,759	1.99	914,585	1,216,118
YR155	1,928,071	1,284,972	641,497	2	792,662	1,135,409
YR157	2,145,065	1,433,621	709,442	2.02	1,084,613	1,060,452
YR158	1,968,391	1,317,598	649,175	2.03	880,325	1,088,066
YR164	1,738,195	1,157,378	579,499	2	609,132	1,129,063
YR166	2,064,851	1,376,138	686,755	2	976,916	1,087,935
YR168	1,969,252	1,307,374	660,117	1.98	819,031	1,150,221
YR172	2,059,631	1,368,028	689,719	1.98	881,447	1,178,184
YR173	1,793,748	1,192,879	599,395	1.99	691,693	1,102,055
YR174	1,916,449	1,274,556	640,221	1.99	787,217	1,129,232
YR178	2,066,407	1,374,373	690,098	1.99	954,701	1,111,706
YR180	1,923,720	1,279,645	642,287	1.99	832,923	1,090,797
YR181	1,957,196	1,302,940	652,403	2	882,227	1,074,969
YR183	1,852,783	1,232,997	618,177	1.99	771,622	1,081,161
YR184	1,845,522	1,230,174	613,805	2	778,334	1,067,188
YR186	1,952,926	1,292,992	658,473	1.96	629,885	1,323,041
YR187	1,907,989	1,270,589	635,766	2	802,044	1,105,945
YR189	1,815,628	1,212,446	601,752	2.01	668,388	1,147,240
YR192	1,841,093	1,225,660	613,814	2	746,482	1,094,611
YR193	1,909,129	1,270,531	636,997	1.99	762,875	1,146,254
YR195	1,851,055	1,232,718	616,778	2	768,921	1,082,134
YR196	1,897,396	1,265,202	630,569	2.01	806,469	1,090,927
YR198	1,885,235	1,252,757	630,740	1.99	851,934	1,033,301
YR199	1,785,959	1,189,425	595,129	2	690,739	1,095,220
YR201	1,809,583	1,203,676	604,480	1.99	648,740	1,160,843
YR203	1,891,162	1,255,809	633,705	1.98	770,193	1,120,969
YR204	1,948,145	1,300,458	645,913	2.01	921,075	1,027,070
YR205	1,793,382	1,199,189	592,741	2.02	731,447	1,061,935
YR208	1,815,482	1,212,970	600,836	2.02	834,808	980,674
YR209	1,781,271	1,183,723	596,051	1.99	730,241	1,051,030
YR211	1,818,444	1,213,295	603,516	2.01	805,829	1,012,615
YR212	1,799,271	1,198,941	598,748	2	771,813	1,027,458
YR214	1,901,559	1,267,564	632,299	2	832,419	1,069,140
YR215	1,848,226	1,233,907	612,688	2.01	808,487	1,039,739
YR217	1,816,638	1,209,206	605,921	2	762,206	1,054,432
YR218	1,921,265	1,284,862	634,650	2.02	872,072	1,049,193
YR223	2,321,894	1,541,498	778,266	1.98	1,045,577	1,276,317
YR224	1,846,435	1,229,870	615,013	2	736,085	1,110,350
YR226	1,881,463	1,253,934	625,896	2	806,906	1,074,557
YR227	1,666,384	1,112,587	552,413	2.01	669,714	996,670
YR229	1,796,434	1,198,793	596,098	2.01	746,212	1,050,222

YR230	1,717,061	1,144,055	571,612	2	625,849	1,091,212
YR235	1,895,288	1,267,133	626,499	2.02	860,066	1,035,222
YR237	1,842,268	1,229,021	611,598	2.01	824,345	1,017,923
YR238	2,076,813	1,394,227	680,575	2.05	1,083,627	993,186
YR242	1,762,601	1,175,700	585,496	2.01	657,191	1,105,410
YR245	1,737,646	1,158,828	577,475	2.01	667,453	1,070,193
YR66	1,655,168	1,106,742	547,004	2.02	720,293	934,875
YR75	1,875,271	1,255,122	618,540	2.03	814,546	1,060,725
YR76	1,848,754	1,236,283	610,828	2.02	842,349	1,006,405
YR79	1,877,347	1,260,783	614,980	2.05	850,835	1,026,512

Notes:

Sample ID：Sample number.

YR：Yunling Goat.

NBY：Nubian Goat.

SNP Number：The number of single nucleotide polymorphisms, in this table, represents the number of SNPs between the sample genome and the reference genome.

Transition：Number of transition SNPs.

Transversion：Number of transversion SNPs.

Ti/Tv：Ratio of transition SNP and transversion SNP.

Heterozygosity Number：The total number of SNP loci of heterozygous typing.

Homozygosity Number：The total number of SNP loci of homozygous typing.

Table S4. Statistical table of InDel.

Sample ID	Insertion Number	Deletion Number	Heterozygosity Number	Homozygosity Number
NBY01	145,140	132,745	129,479	148,406
NBY05	139,944	128,553	123,438	145,059
NBY07	139,275	127,590	124,338	142,527
NBY08	145,828	134,835	131,000	149,663
NBY10	143,157	131,756	129,122	145,791
NBY100	151,266	140,089	143,312	148,043
NBY101	141,041	129,156	126,919	143,278
NBY103	151,208	139,807	139,688	151,327
NBY105	156,713	145,404	144,760	157,357
NBY107	152,810	139,471	139,940	152,341
NBY109	154,765	142,059	139,683	157,141
NBY11	148,192	135,904	117,748	166,348
NBY110	149,830	137,985	136,175	151,640
NBY112	153,425	141,042	137,437	157,030
NBY114	161,799	147,524	143,499	165,824
NBY115	142,228	130,397	118,641	153,984
NBY117	151,737	138,931	137,667	153,001
NBY118	151,638	139,481	136,882	154,237
NBY120	136,436	125,229	121,410	140,255
NBY122	162,971	149,337	144,113	168,195
NBY124	156,244	144,043	144,710	155,577
NBY125	160,724	148,159	148,028	160,855
NBY13	160,197	147,894	148,017	160,074
NBY14	140,380	129,223	126,274	143,329
NBY16	143,966	132,187	130,985	145,168
NBY17	145,030	133,159	130,427	147,762
NBY19	144,260	132,839	130,163	146,936
NBY20	140,015	128,685	127,128	141,572
NBY22	146,242	133,623	127,612	152,253
NBY23	145,103	133,281	129,567	148,817
NBY27	132,276	122,236	113,162	141,350
NBY29	139,281	128,071	126,082	141,270
NBY32	150,657	138,400	135,171	153,886
NBY35	144,608	134,029	129,782	148,855
NBY36	139,394	128,846	126,158	142,082
NBY38	142,224	130,912	127,605	145,531
NBY40	150,251	138,138	135,186	153,203
NBY42	151,486	138,431	134,544	155,373
NBY44	145,551	133,110	129,647	149,014
NBY48	147,891	136,385	132,481	151,795
NBY52	134,275	122,177	115,433	141,019
NBY53	140,212	128,235	124,130	144,317
NBY55	144,503	133,442	127,931	150,014
NBY56	129,775	119,014	114,011	134,778
NBY58	133,430	122,586	114,568	141,448
NBY59	153,694	140,717	140,527	153,884
NBY61	146,799	134,049	127,570	153,278
NBY63	141,268	129,612	125,016	145,864
NBY69	124,261	113,089	105,647	131,703
NBY71	142,552	130,104	124,327	148,329
NBY73	153,276	141,584	139,221	155,639
NBY83	143,865	131,854	128,630	147,089
NBY85	130,913	120,023	114,405	136,531
NBY87	142,667	130,647	127,115	146,199

NBY88	149,602	138,122	135,305	152,419
NBY90	154,461	143,332	141,745	156,048
NBY92	163,781	151,132	152,616	162,297
NBY94	155,086	143,451	145,303	153,234
NBY95	151,561	140,300	139,430	152,431
NBY97	145,867	134,526	133,686	146,707
YR123	143,416	131,342	121,410	153,348
YR131	138,671	125,090	113,358	150,403
YR132	140,410	127,495	111,734	156,171
YR135	142,575	129,364	111,465	160,474
YR140	161,474	148,004	140,200	169,278
YR142	152,161	139,995	130,074	162,082
YR143	140,704	126,563	105,650	161,617
YR147	141,265	127,769	109,454	159,580
YR148	144,914	131,985	115,623	161,276
YR152	175,956	161,768	149,714	188,010
YR155	158,361	144,090	131,282	171,169
YR157	171,626	160,154	161,591	170,189
YR158	159,799	146,017	139,103	166,713
YR164	142,751	129,478	107,556	164,673
YR166	166,129	153,190	149,850	169,469
YR168	159,161	146,554	130,245	175,470
YR172	169,871	155,911	144,467	181,315
YR173	147,517	133,766	117,026	164,257
YR174	157,832	144,340	130,180	171,992
YR178	166,939	154,367	148,571	172,735
YR180	155,381	143,081	131,322	167,140
YR181	158,639	145,749	137,819	166,569
YR183	150,106	137,578	123,954	163,730
YR184	149,497	136,950	125,028	161,419
YR186	168,522	152,527	124,992	196,057
YR187	155,714	142,180	130,335	167,559
YR189	151,934	138,383	119,756	170,561
YR192	151,038	137,747	122,665	166,120
YR193	158,604	144,585	128,662	174,527
YR195	151,627	138,105	125,043	164,689
YR196	155,450	141,551	130,566	166,435
YR198	150,792	138,571	130,417	158,946
YR199	146,815	133,170	117,349	162,636
YR201	150,622	136,502	116,491	170,633
YR203	155,029	141,461	127,327	169,163
YR204	156,747	143,666	141,196	159,217
YR205	145,852	133,700	119,357	160,195
YR208	142,550	131,720	124,646	149,624
YR209	144,072	131,071	116,753	158,390
YR211	144,835	132,723	124,218	153,340
YR212	143,949	131,428	120,633	154,744
YR214	152,994	140,617	130,666	162,945
YR215	149,193	136,688	125,788	160,093
YR217	147,313	133,921	121,550	159,684
YR218	154,011	141,981	135,559	160,433
YR223	195,895	179,139	174,677	200,357
YR224	152,008	137,591	123,864	165,735
YR226	152,143	139,821	127,125	164,839
YR227	134,677	122,259	108,520	148,416
YR229	146,222	133,842	120,757	159,307
YR230	141,291	127,437	108,156	160,572

YR235	152,429	139,267	133,669	158,027
YR237	147,685	134,721	127,705	154,701
YR238	155,805	143,057	142,349	156,513
YR242	146,117	132,394	114,490	164,021
YR245	141,926	128,919	112,454	158,391
YR66	130,950	119,801	110,776	139,975
YR75	151,142	137,988	129,811	159,319
YR76	146,914	134,315	128,625	152,604
YR79	152,186	138,452	133,276	157,362

Notes:

Sample ID：Sample number.

YR：Yunling Goat.

NBY：Nubian Goat.

SNP Number：The number of single nucleotide polymorphisms, in this table, represents the number of SNPs between the sample genome and the reference genome.

Transition：Number of transition SNPs.

Transversion：Number of transversion SNPs.

Ti/Tv：Ratio of transition SNP and transversion SNP.

Heterozygosity Number：The total number of SNP loci in heterozygous typing.

Homozygosity Number：Total number of SNP loci in homozygous typing.

Insertion Number：The number of insertion sites.

Deletion Number：The number of deletion sites.

Heterozygosity Number：The number of InDel loci of heterozygous type.

Homozygosity Number：The number of InDel loci of homozygous type.
